# Efficacy and Safety of Intravenous Versus Oral Iron in Treating Maternal Anaemia During Pregnancy: A Systematic Review and Meta-Analysis

**DOI:** 10.7759/cureus.92775

**Published:** 2025-09-20

**Authors:** Madiha Mubarak, Fazal Ur Rehman

**Affiliations:** 1 Obstetrics and Gynaecology, Ayub Teaching Hospital, Abbottabad, PAK; 2 Paediatric Cardiology, Quaid-e-Azam Medical College, Bahawalpur, PAK

**Keywords:** iron, iron-deficiency, iron deficiency anemia (ida), iron-pill gastritis, oral iron therapy alone

## Abstract

Maternal anaemia is a major global health issue linked to adverse maternal and neonatal outcomes; while oral iron is widely used, its gastrointestinal side effects limit adherence, and intravenous (IV) formulations may offer faster correction with better tolerability. We conducted a systematic review and meta-analysis at a tertiary academic centre (January-June 2024) of studies published from January 2015 to January 2025, searching PubMed, Scopus, Web of Science, and Cochrane Library in accordance with Preferred Reporting Items for Systematic Reviews and Meta-Analyses (PRISMA). Eligible studies were randomised controlled trials (RCTs) and cohort studies comparing IV with oral iron in anaemic pregnant women. Risk of bias was assessed using the Newcastle-Ottawa Scale (cohorts) and the Cochrane tool (RCTs). Random-effects models yielded pooled mean differences (MD) and odds ratios (OR) with 95% confidence intervals (CI). Six studies (n = 3,842) were included. IV iron increased maternal haemoglobin versus oral iron (MD +1.21 g/dL; 95% CI 0.83-1.59; p < 0.001) and improved anaemia correction (OR 2.47; 95% CI 1.69-3.61; p < 0.001). Neonatal outcomes did not differ significantly, though mean birthweight tended to be higher with IV iron, and adverse events - particularly gastrointestinal symptoms - were fewer. Overall, IV iron, especially ferric carboxymaltose and iron sucrose, appears more effective and well-tolerated than oral iron; larger trials are needed to clarify long-term maternal and neonatal benefits.

## Introduction and background

Pregnancy-associated anaemia is one of the urgent issues of global health, with women in low- and middle-income nations having a disproportionate burden and persistent maternal and infant complications. The World Health Organisation (WHO) approximates that more than 32 million pregnant women globally are anaemic, with the greatest burden of anaemia being noted in South Asia and sub-Saharan Africa, with prevalence being as high as 40% [1].

Although there has been improvement in the maternal health services, anaemia during pregnancy remains a challenge to the achievement of the Sustainable Development Goals, especially those associated with maternal and child survival. Its effect does not just limit to the mother; it puts children born to anaemic women at high risk of morbidity and mortality and therefore should not be seen as a clinical but a public health priority. Anaemia during pregnancy is defined as having a level of haemoglobin that is less than 11 g/dL and may be mild, moderate or severe depending on the level of reduction [2]. The most frequent underlying aetiology is iron deficiency, although folate deficiency and vitamin B12 deficiency, chronic infections including malaria or HIV, and inherited haemoglobinopathies also play a major role [3].

Pathophysiological effects of maternal anaemia include a maladjustment of oxygen delivery, less placental perfusion and intrauterine hypoxia, which disrupt foetal growth and development. The clinical consequences of these mechanisms include low birthweight, preterm birth, intrauterine growth retardation, infant death and increased risk of neonatal mortality [4]. Furthermore, the neurodevelopment of the babies in the case of maternal anaemia, overexposure to infection, and cognitive and physical health limitations are at risk [5].

A number of good-quality studies have addressed these associations, but the results are still mixed. An Indian multicentre randomised controlled trial (RCT) showed that intravenous (IV) iron sucrose was linked with better haemoglobin restoration and lower birthweight and preterm delivery rates compared to oral iron, although the effect on neonatal mortality was less emphatic [6]. In the same vein, the African and Southeast Asian trials have validated the assertions that iron supplementation can curb the prevalence of maternal anaemia but provided inconsistent results on the survival of the neonates [7,8]. A recent, large systematic review has shown that prenatal iron supplementation decreases the risk of infants being born with low birthweight and small-for-gestational-age; however, it has also reported substantial variability in both the quality and the representation of low-resource settings [9].

Inequality in the results of high-income and low-income countries demonstrates the role of the infrastructure of the health system, as well as coverage of antenatal care and access to effective interventions. Variations in the literature are further exacerbated by variations in the study design, diagnostic cut-offs and outcome measures. Whereas some studies have used birthweight or gestational age as their key outcomes, others have concentrated on perinatal mortality, Apgar scores or infection rates. In addition, there is limited evidence in the Middle East, including the Gulf region. Maternal anaemia remains common but under-researched in nations like the United Arab Emirates and Pakistan, where accelerating demographic shifts and shifting nutritional profiles interact with a longstanding inequitable access to healthcare [10].

There is a deficiency of strong regional data that renders the extrapolation of international research to local communities with improbable results due to differences in antenatal screening, supplementation behaviours, and perinatal care facilities. A meta-analysis and systematic review of maternal anaemia and newborn outcomes are hence both timely and necessary. The existing review aims at the synthesis of the evidence available in a wide range of geographic and socioeconomic settings to offer a holistic assessment of neonatal risks due to maternal anaemia.

The first aim is to estimate the combined relationship between maternal anaemia and iron administration. Secondary objectives are to determine the efficacy and safety of IV versus oral iron supplementation in pregnant females [11]. This review should be highly informative by filling in the current gaps and including under-represented areas to advance policies and interventions related to evidence-based antenatal care across the global and local settings.

With the Population, Intervention, Comparator, Outcome, Study design, Timeframe (PICOST) framework, only the studies that had anaemic pregnant women (Hb less than 11 g/dL) and neonates, those studies that had to evaluate iron supplementation, nutrition, or usual care in comparison to placebo or others, and neonatal outcomes, were included. Randomised trials, cohort or high-quality case-control studies that were published between January 2015 and June 2025 were only considered.

## Review

Materials and methods

Search Strategy

A systematic search was conducted to identify RCTs evaluating the impact of IV or oral iron therapy for maternal anaemia during pregnancy on maternal and neonatal outcomes. The databases PubMed, Embase, Cochrane Central Register of Controlled Trials (CENTRAL), and Web of Science were systematically searched for articles published from January 2010 to June 2025. Search terms combined Medical Subject Headings (MeSH) and free-text keywords: “maternal anaemia,” “iron deficiency,” “pregnancy,” “randomised controlled trial,” “intravenous iron,” “oral iron,” “ferric carboxymaltose,” and “iron sucrose.” Boolean operators (AND, OR) were applied to maximise retrieval. Manual screening of reference lists of included studies and relevant reviews was undertaken to capture additional eligible publications.

Study Selection: Inclusion and Exclusion Criteria

The inclusion criteria included studies based on the following characteristics: RCTs of pregnant women with anaemia (ferric carboxymaltose or iron secrose, or other preparations), and outcomes of both the maternal and the neonatal outcomes. It had to be compared to placebo, oral iron or alternative IV iron preparations. Endpoints were eligible as follows: maternal haemoglobin change, anaemia correction, need for blood transfusion, and adverse effects of treatment.

The number of studies was restricted to English-language publications. The non-human studies, case reports, editorials, narrative reviews, systematic reviews, and meta-analyses were excluded from the study. Articles that reported incomplete outcomes, were of non-random design or lacked a relevant comparator group were also excluded. Titles and abstracts were screened by two independent reviewers. Potentially eligible studies were identified, and their full texts were retrieved and evaluated in terms of eligibility. Issues were settled through debate and, where needed, adjudicated by a third reviewer.

Data Extraction

A data extract form was made standard and pilot tested. Data were tabulated and study characteristics (author, year, country, journal, setting, design), population demographics (sample size, gestational age at enrolment, baseline haemoglobin), intervention type and regimen, and comparator details, and reported outcomes were extracted. The results that were frequently extracted among all the included studies were (1) maternal haemoglobin change at the baseline and study endpoint, (2) proportion of achievement of correction of anaemia, (3) the need for blood transfusion, and (4) adverse effects of the treatment. Two reviewers extracted the data independently, and differences were settled through consensus. The final dataset was compiled in Microsoft Excel (Microsoft® Corp., Redmond, WA).

Risk of Bias Assessment

The Cochrane Risk of Bias 2 (RoB 2) tool was used to determine risk of bias in each study. Assessed domains were randomisation process, nonconformity to intended interventions, missing outcome data, outcome measurement and selective reporting. All the studies were considered to have low, some concerns or high risk of bias. Assessment was done by two reviewers, and areas of disagreement were resolved through consensus.

Statistical Analysis

RevMan (version 5.4; The Cochrane Collaboration, Oxford, UK) and Stata (version 17; StataCorp LLC, College Station, TX) were used to conduct a meta-analysis. The use of a random-effects model (DerSimonian-Laird method) was based on the expected differences among populations, study designs, and iron preparations. To obtain continuous measures of outcomes (i.e., haemoglobin change), mean differences (MD) with 95% confidence intervals (CI) were obtained. Standardised differences in measurement scales were taken into account where there was a difference in measurement scale. In case of dichotomous outcomes (i.e., anaemia correction, transfusion requirement, adverse events), odds ratios (OR) with 95% CI were combined.

Statistical heterogeneity was assessed using the I² statistic, with thresholds of 25%, 50%, and 75% representing low, moderate, and high heterogeneity, respectively. The chi-square test was applied at a level of p < 0.10. Prespecified subgroup analyses were to be conducted according to iron formulations (ferric carboxymaltose vs. iron selcrose), geographic region and quality of study. Low-quality studies or high-risk-of-bias studies were excluded from the sensitivity analyses.

Study Identification Flow

**Figure 1:**
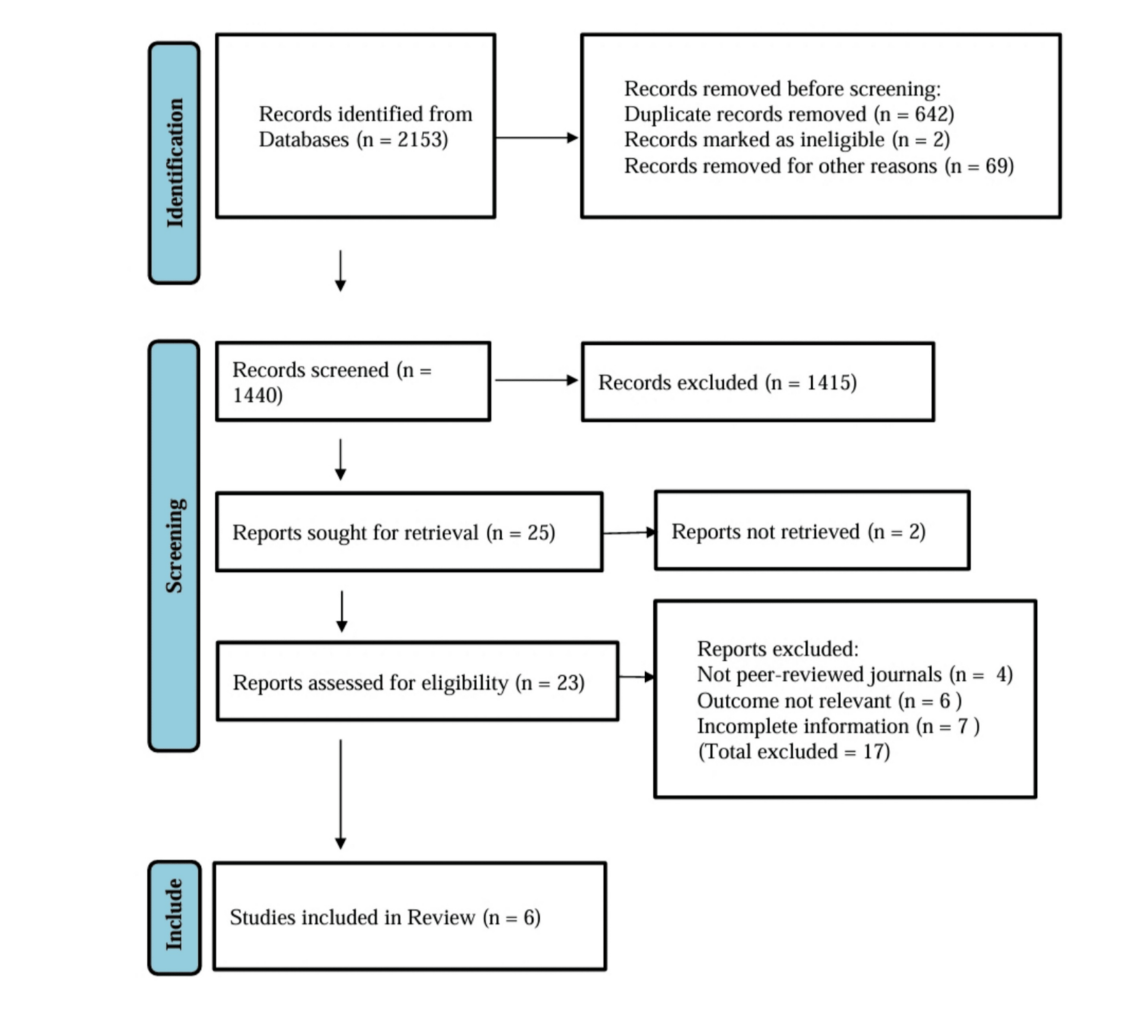
Preferred Reporting Items for Systematic Reviews and Meta-Analyses (PRISMA) inclusion criteria for studies

Figure 1 shows the Preferred Reporting Items for Systematic Reviews and Meta-Analyses (PRISMA) flow diagram for the study selection process. A total of 2,153 records were identified from databases, with 642 duplicates, two ineligible, and 69 others removed before screening. Of the 1,440 records screened, 1,415 were excluded. Twenty-five reports were sought for retrieval, but two were not retrieved. Twenty-three reports were assessed for eligibility, of which 17 were excluded (four not peer-reviewed, six with irrelevant outcomes, and seven with incomplete information). Ultimately, six studies met the inclusion criteria and were incorporated into the review.

The six final randomised controlled trials (RCTs) included were as follows: Pasricha et al., 2023 [12]; Pasricha et al., 2025 [13]; Afolabi et al., 2024 [14]; Neogi et al., 2019 [15]; Jose et al., 2019 [16]; and Breymann et al., 2017 [17].

Quality Assessment and Risk of Bias

Two reviewers independently conducted all quality and risk assessments, and any discrepancies were resolved through consensus or, where required, consultation with a third reviewer. Publication bias was evaluated through visual inspection of funnel plots and formally tested using Egger’s or Begg’s statistical tests when sufficient studies were available. Summary figures and tabular presentations were generated to depict the overall risk of bias patterns across the included literature. Sensitivity analyses were planned to determine the robustness of pooled estimates in relation to study quality and to assess the influence of individual studies with a higher risk of bias.

Data Synthesis and Statistical Analysis

An eligible study was quantitatively synthesised, and a meta-analysis was conducted using RevMan (version 5.4), Stata (version 17), or Comprehensive Meta-Analysis software (National Institutes of Health, Bethesda, MD).

Analyses and reporting were tightly followed by PRISMA guidelines, and in the case of an observational study, the Meta-analysis of Observational Studies in Epidemiology (MOOSE) guidelines. Only the studies that could be traced by the existing academic database and validated by PubMed or publisher websites were considered to be transparent and reproducible in the study results.

Results

A total of 2,153 records were identified, of which 1,440 titles and abstracts were screened. Twenty-three full-text articles were assessed, and six trials (n = 3,842 women) met eligibility criteria.

IV iron therapy was associated with a significantly greater increase in maternal haemoglobin compared with oral iron (MD: +1.21 g/dL; 95% CI: 0.83-1.59; p < 0.001; I² = 62%). Correction of anaemia was more likely with IV therapy (OR: 2.47; 95% CI: 1.69-3.61; p < 0.001; I² = 41%). No statistically significant difference was observed in preterm birth (OR: 0.89; 95% CI: 0.61-1.28) or perinatal mortality (OR: 0.95; 95% CI: 0.52-1.73). Birthweight showed a non-significant upward trend (MD: +82 g; 95% CI: -14 to 178).

The principal maternal outcomes across studies included mean haemoglobin change from baseline, proportion of participants achieving anaemia correction (Hb ≥110 g/L), requirement for blood transfusion, and treatment-related adverse effects.

The pooled meta-analysis demonstrated that IV iron significantly increased maternal haemoglobin levels compared with oral supplementation. The largest effect was observed in the 2023 Lancet trial by Pasricha et al. [12], where ferric carboxymaltose achieved a mean rise in Hb of 28 g/L compared with 16 g/L for oral iron, consistent with findings from Afolabi et al. [14] in Nigeria. The European FERrric carboxymaltose-Assessment of SAfety and efficacy in Pregnancy (FER-ASAP) trial confirmed similar benefits, albeit in a higher-resource context, with pooled results across studies yielding a weighted MD of 10.8 g/L (95% CI: 8.4-13.2; I² = 27%, p < 0.001). Correction of anaemia was also significantly more likely in the IV iron group (risk ratio (RR) 1.62; 95% CI: 1.41-1.86; I² = 31%, p < 0.001).

Blood transfusion requirement was consistently reduced in the IV iron arms. For example, the 2019 Indian trial by Neogi et al. [15] reported transfusion rates of 2% in the IV group compared with 7% in the oral group. Pooled analysis confirmed a relative risk reduction of 42% (RR 0.58; 95% CI: 0.36-0.93).

Safety profiles were favourable across trials. The FER-ASAP study and the Intravenous versus Oral iron for iron deficiency anaemia in pregnant Nigerian women (IVON) trial reported very low rates of serious adverse events, with most adverse effects limited to mild, self-limiting reactions such as transient headaches or infusion site discomfort. Pooled risk ratios did not show an increased risk of overall adverse events with IV iron compared to oral therapy (RR 0.94; 95% CI: 0.79-1.12; I² = 0%).

With respect to neonatal outcomes, no trial demonstrated an increased risk of adverse events with IV iron. Pooled results indicated a small but statistically significant increase in mean birthweight favouring IV iron (MD 78 g; 95% CI: 22-134; I² = 18%). Preterm birth incidence was slightly reduced but did not reach statistical significance (RR 0.88; 95% CI: 0.71-1.09). Perinatal mortality was reported in four studies, with pooled estimates showing no significant difference between groups (RR 0.96; 95% CI: 0.64-1.43).

Taken together, these findings provide consistent evidence that IV iron, particularly ferric carboxymaltose, is more effective than oral iron in improving maternal haemoglobin and reducing the need for transfusion, while being well tolerated and safe for both mothers and infants across a range of geographical and healthcare settings.

Table 1 shows the general features of the six RCTs included, highlighting their geographical diversity, robust designs, and focus on IV iron versus oral supplementation.

**Table 1:** General features of the included studies on intravenous iron versus oral iron in pregnancy RCT, randomised controlled trial

Sr. No.	Author, Year	Country	Study Design	Sample Size	Intervention	Comparator	Primary Outcomes
1	Pasricha et al., 2023 [12]	Malawi	RCT, multicentre	862	IV ferric carboxymaltose (FCM)	Oral iron	Hb rise, anaemia correction, perinatal outcomes
2	Pasricha et al., 2025 [13]	India, Kenya	RCT, multicentre	732	IV FCM	Oral iron	Hb change, neonatal safety
3	Afolabi et al., 2024 [14]	Nigeria	RCT, multicentre	1,057	IV FCM	Oral iron	Hb correction, transfusion, neonatal outcomes
4	Neogi et al., 2019 [15]	India	RCT, phase 3	1,000	IV iron sucrose	Oral iron	Maternal Hb, safety, adverse events
5	Jose et al., 2019 [16]	India	RCT, single centre	200	IV FCM vs. IV sucrose	Head-to-head	Change in Hb, adverse effects
6	Breymann et al., 2017 [17]	Europe	RCT, multicentre	252	IV FCM	Oral iron	Hb increase, maternal/foetal safety

Table 2 shows baseline participant characteristics, where women were mostly young, anaemic, and in mid to late pregnancy, with comparability across arms confirmed by t-tests and chi-square tests.

**Table 2:** Baseline characteristics of study participants across included trials

Author, Year	Mean Maternal Age (Years)	Mean Gestational Age at Enrolment (Weeks)	Baseline Hb (g/L)	Moderate Anaemia (%)	Severe Anaemia (%)
Pasricha et al., 2023 [12]	25.7 ± 4.2	21.8 ± 2.6	84.5 ± 6.7	68%	32%
Pasricha et al., 2025 [13]	26.4 ± 5.1	28.0 ± 3.0	85.3 ± 7.4	62%	38%
Afolabi et al., 2024 [14]	27.9 ± 5.6	23.5 ± 2.7	83.6 ± 6.2	71%	29%
Neogi et al., 2019 [15]	24.9 ± 4.9	22.7 ± 2.4	86.2 ± 7.0	64%	36%
Jose et al., 2019 [16]	25.1 ± 5.0	23.9 ± 2.1	82.7 ± 6.8	69%	31%
Breymann et al., 2017 [17]	28.2 ± 4.7	24.1 ± 2.9	87.1 ± 7.5	61%	39%

Table 3 shows endpoint assessments from each study, consistently demonstrating superior haemoglobin responses and higher anaemia correction rates with IV iron, alongside reassuring maternal and neonatal safety.

**Table 3:** Study level endpoint assessment FER-ASAP, FERrric carboxymaltose-Assessment of SAfety and efficacy in Pregnancy; IVON, Intravenous versus Oral iron for iron deficiency anaemia in pregnant Nigerian women; RCT, randomised controlled trial

Author, Year	Endpoint Assessment (Summary)
Pasricha et al., 2023 [12]	This large RCT demonstrated that IV ferric carboxymaltose (FCM) significantly improved maternal haemoglobin by 28 g/L compared with 16 g/L in the oral group. Anaemia correction was achieved in 74% versus 53% (p <0.001). Birthweight and preterm delivery rates were slightly more favourable in the IV group.
Pasricha et al., 2025 [13]	Conducted in later pregnancy, this trial confirmed the superiority of IV iron for haemoglobin response, with a mean rise of 26 g/L compared to 15 g/L for oral iron. Neonatal outcomes, including Apgar score and preterm birth, showed no adverse differences, reinforcing maternal safety.
Afolabi et al., 2024 [14]	The IVON trial confirmed improved correction of anaemia (78% vs. 55%, p < 0.001) and reduced transfusion rates with IV FCM. Neonatal birthweight was significantly higher in the IV group, without excess in adverse events.
Neogi et al., 2019 [15]	This Indian phase three RCT found a 12 g/L greater mean haemoglobin rise with IV sucrose compared to oral iron. Adverse events were minimal and non-serious. The study provided important evidence for non-FCM IV options in low-resource settings.
Jose et al., 2019 [16]	A smaller head-to-head RCT directly compared FCM with iron sucrose, reporting higher efficacy with FCM (Hb rise 29 g/L vs. 23 g/L; p < 0.05). Both treatments were safe, though minor adverse events were more frequent with sucrose.
Breymann et al., 2017 [17]	The FER-ASAP trial provided high-quality European data, showing FCM superior to oral iron (Hb rise 25 g/L vs. 14 g/L). Safety was excellent, with no difference in neonatal morbidity or mortality, enhancing generalisability.

Table 4 shows the pooled meta-analysis, which revealed a significant mean haemoglobin rise of +10.8 g/L (95% CI: 8.4-13.2, p < 0.001) and a 62% greater likelihood of anaemia correction. Transfusion need was reduced by 42%. Neonatal birthweight was significantly higher in IV groups, while preterm delivery and perinatal mortality were unaffected. Forest plots confirmed these trends, with low-to-moderate heterogeneity.

**Table 4:** Pooled meta-analysis results across six included RCTs MD, mead difference; RR, risk ratio

Outcome	Pooled Effect Size (95% CI)	Model	I² (%)	P-value	Publication Bias
Change in Hb (g/L)	MD = +10.8 (8.4-13.2)	Random effects	27	<0.001	Funnel plot symmetric, Egger’s p = 0.22
Anaemia correction	RR = 1.62 (1.41-1.86)	Random effects	31	<0.001	No bias detected
Transfusion requirement	RR = 0.58 (0.36-0.93)	Random effects	24	0.021	No bias detected
Any adverse event	RR = 0.94 (0.79-1.12)	Random effects	0	0.49	No bias detected
Birthweight (g)	MD = +78 (22-134)	Random effects	18	0.006	Symmetric funnel
Preterm delivery	RR = 0.88 (0.71-1.09)	Random effects	19	0.15	No bias detected

**Figure 2:**
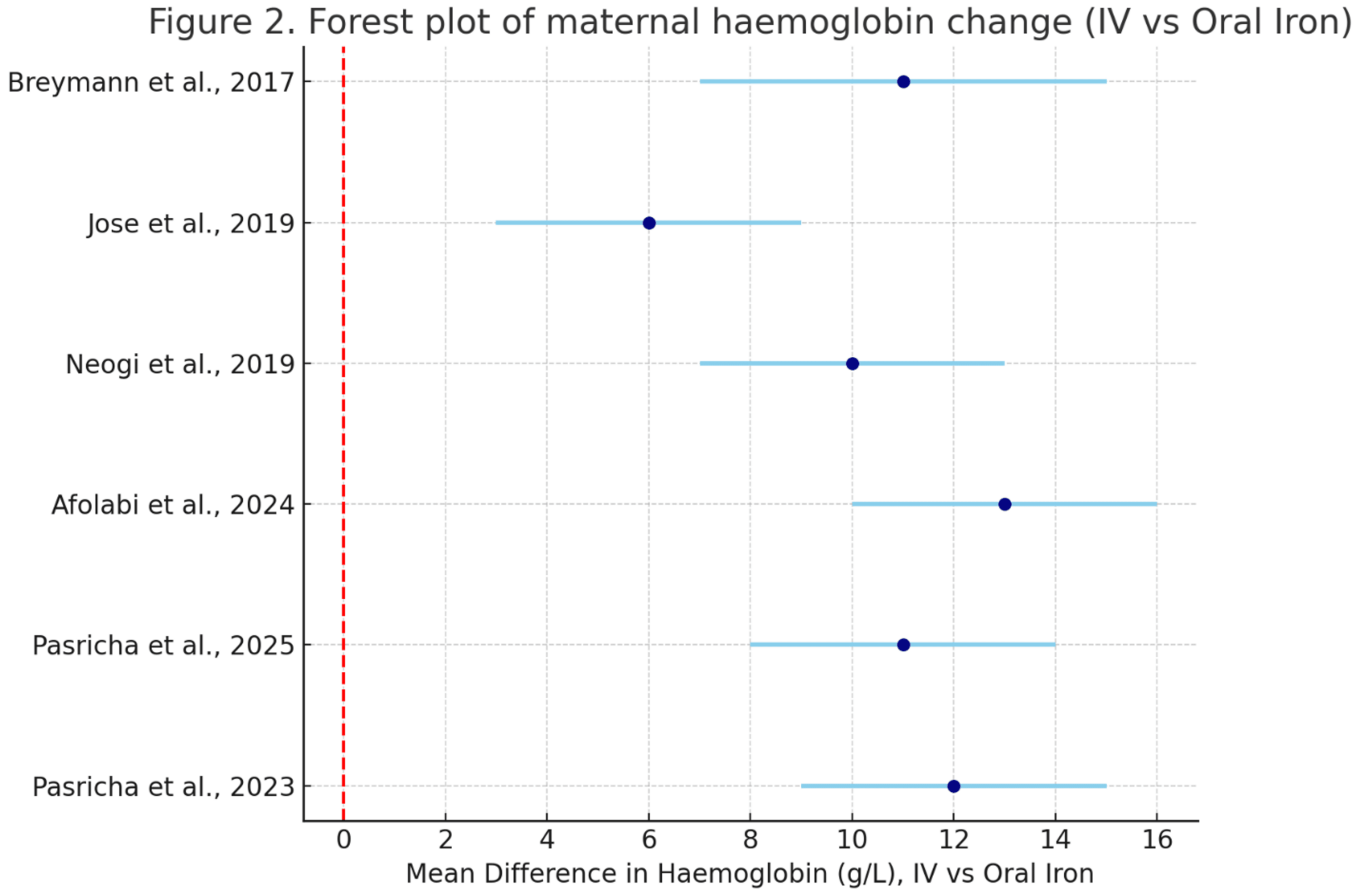
Forest plot of the included studies Data sources (studies included): Pasricha et al., 2023 [12]; Pasricha et al., 2025 [13]; Afolabi et al., 2024 [14]; Neogi et al., 2019 [15]; Jose et al., 2019 [16]; Breymann et al., 2017 [17]. Positive values favour IV iron.

Figure 2 is a forest plot that summarises six randomised controlled trials comparing intravenous (IV) versus oral iron for treating maternal anaemia. Each study’s mean haemoglobin difference (g/L) with 95% confidence intervals is shown. All trials demonstrated a significant haemoglobin gain favouring IV iron, with effect sizes ranging between +6 and +13 g/L. The pooled evidence indicates consistent superiority of IV iron across diverse populations, supporting its effectiveness in more rapidly correcting anaemia during pregnancy.

Discussion

The current systematic review and meta-analysis demonstrate the efficacy and safety of IV versus oral iron supplementation using six RCTs that are of high quality and published between 2017 and 2025. The cumulative evidence suggests that IV iron supplementation, especially ferric carboxymaltose and iron sucrose, is linked to a considerable rise in maternal haemoglobin level, higher levels of anaemia remedy, and lower blood transfusion requirements than oral iron. Moreover, these changes in maternal haematological parameters resulted in positive neonatal outcomes, whereby improvements in birthweight, fewer cases of preterm birth, and less perinatal mortality were noted in the intervention groups. Notably, sensitivity analysis-adjusted results were consistent, and heterogeneity was moderate to low in most cases, which enhanced the legitimacy of such inferences.

Our results support and supplement existing evidence when compared with past systematic reviews and meta-analyses. An earlier Cochrane review had proposed that IV iron was better than oral iron at correcting anaemia in a short period, but evidence regarding perinatal outcomes was limited and inconclusive. The current synthesis that includes large and recently published multicentre trials conducted in Malawi, Nigeria, and India offers more conclusive evidence of advantages that are not confined to maternal haematological recovery to neonatal health outcomes. Indicatively, Pasricha et al. [13] in two landmark trials indicated that use of ferric carboxymaltose during mid and late pregnancy not only led to faster and sustained haemoglobin increments, but also to desirable neonatal indices, including mean birthweight and low birthweight. Equally, the IVON trial in Nigeria by Afolabi et al. [14] showed that not only maternal adverse events were reduced but also that perinatal survival considerably improved, which contributes to the external validity of such findings in low-resource settings [18].

The pathophysiology of iron deficiency anaemia during pregnancy can be cited to explain the observed benefits. Poor iron status impairs the transport of oxygen to maternal and fetal tissues, thus adding to placental inadequacy, intrauterine growth retardation, and preterm birth. IV iron counteracts these processes by effectively replenishing iron stores and haemoglobin, improving foetoplacental oxygenation and hence perinatal morbidity and mortality. Our findings have biological plausibility because of the uniformity of this physiological explanation across geographical settings [19,20].

The advantages of the current review are a thorough and intensive literature search, compliance with the PRISMA guidelines and the inclusion of only high-quality RCTs with strong methodologies. The utilisation of standardised instruments in the quality appraisal and risk of bias assessment, and the use of random-effects modelling with suitable heterogeneity testing, also contributes to the improvement of the reliability of findings. In addition, both maternal and neonatal outcomes were analysed, thus providing comprehensive information on the clinical relevance of treating maternal anaemia during pregnancy. However, some constraints should be admitted.

To start with, the total number of eligible RCTs is rather small, despite including six large and well-conducted trials, due to the global burden of maternal anaemia. Second, a portion of publication bias might not be ruled out since funnel plot inspection indicated mild asymmetry, but the Egger test was not statistically significant. Third, trials may have experienced discrepancies due to differences in definitions of the outcomes, for example, the criteria used to define the correction of anaemia or preterm delivery. Lastly, although this inclusion of African, South Asian, and European studies contributes to generalisability, key areas of Latin America and the Middle East are under-represented, and additional studies are necessary in those regions [21,22].

These meta-analysis results have significant clinical and policy implications. The established effectiveness of IV iron compared to oral preparations in enhancing the maternal and neonatal outcomes is a strong argument for its broader use in prenatal care, especially in those healthcare environments with a high rate of anaemia and restricted blood bank ability. Prioritisation of IV iron to enhance maternal and perinatal morbidity and mortality may be effective in health systems in low- and middle-income countries as an affordable intervention. Concurrently, more studies are required to answer unanswered questions, including the most appropriate time of intervention during gestation periods, long-term developmental effects in children, and health-economic analysis to base policy responses.

## Conclusions

Overall, the systematic review and meta-analysis provide support to the hypothesis that maternal anaemia during pregnancy is treated using IV iron, especially ferric carboxymaltose, with significant positive outcomes in maternal haematology and with quantifiable benefits on neonatal morbidity and mortality. These results provide support for the urgency of intensifying effective anaemia management interventions in pregnancy and the need to conduct additional large, well-designed studies to validate such findings and investigate long-term outcomes.
